# Development and validation of a nomogram for decannulation in patients with neurological injury: A prognostic accuracy study

**DOI:** 10.3389/fneur.2022.979160

**Published:** 2022-10-20

**Authors:** Xi Wang, Lu Wang, Zeyu Wang, Yi Sun, Xingdong Liu, Feng Li, Yu Zheng

**Affiliations:** ^1^Department of Neurosurgery, The First Affiliated Hospital of Nanjing Medical University, Nanjing, China; ^2^Department of Rehabilitation Medicine, Qingdao Municipal Hospital, Qingdao, China; ^3^Department of Rehabilitation Medicine, Shanghai Ruijin Rehabilitation Hospital, Shanghai, China; ^4^Department of Neurological Rehabilitation, Wuxi Yihe Rehabilitation Hospital, Wuxi, China; ^5^Department of Rehabilitation Medicine, The First Affiliated Hospital of Nanjing Medical University, Nanjing, China

**Keywords:** neurological injury, tracheostomy, decannulation, predictive factor, nomogram

## Abstract

**Background:**

Tracheostomy is a lifesaving procedure provided for patients with severe neurological injury. However, there is a lack of clarity about whether patients can be decannulated within 6 months in those receiving tracheostomy and what factors can be detected as a predictor for decannulation.

**Objective:**

The objective of this study was to explore predictive factors of decannulation in patients with neurological injury receiving tracheostomy within 6 months and construct a novel nomogram model for clinical diagnosis and treatment.

**Methods:**

This retrospective observational study enrolled patients with neurological injury who were admitted to the ICU of neurosurgical department in the First Affiliated Hospital of Nanjing Medical University between January 2016 and March 2021. Patients were divided into decannulation group and cannulation group according to whether tracheostomy tube removal was performed within 6 months after tracheostomy. Multivariable logistic regression analysis was performed to determine associated risk factors with a bootstrap backward selection process. The nomogram to assess the probability of decannulation at 6 months was constructed based on the regression coefficients of the associated factors and validated by bootstrap resampling. Model performance was measured by examining discrimination (Harrell's C-index), calibration (calibration plots), and utility (Kaplan–Meier curves stratified by the tertile of the predicted probability calculated and subgroup analysis stratified by age and intervention).

**Results:**

A total of 40.1% (147/367) of patients decannulated within 6 months. Significant variables in multivariable logistic regression analysis were age (odds ratio [OR], 0.972; 95% confidence interval [CI], 0.954–0.990), National Institutes of Health Stroke Scale (NIHSS) score (OR, 0.936; 95% CI, 0.911–0.963), early rehabilitation (OR, 5.062; 95% CI, 2.889–8.868), shock (OR, 0.175; 95% CI, 0.058–0.533), and secondary surgery (OR, 0.210; 95% CI, 0.078–0.566). The area under receiver operating characteristic curve estimated with these variables was of 0.793 (95% CI, 0.747–0.838; *P* < 0.001). A nomogram prediction model was constructed to predict the probability of decannulation in tracheostomized patients with a concordance index of 0.788 after internal validation.

**Conclusion:**

We developed a nomogram that can predict the probability of decannulation within 6 months in tracheostomized neurological injury patients. The nomogram, including age, NIHSS scores, early rehabilitation, shock, and secondary surgery, may assist clinicians in estimating patients' prognosis.

## Introduction

Neurological injury includes all those diseases that render patients acutely neurologically devastated. Taken together, neurological injuries can be divided into those with traumatic and nontraumatic causes. The largest groups are stroke and traumatic brain injury (1). Patients with severe neurological injury admitted to intensive care unit (ICU) frequently experience respiratory failure due to loss of airway protective reflexes or decreased respiratory drive and are at risk for pulmonary complications (2). These patients frequently require mechanical ventilation and ultimately tracheostomy. Tracheostomy is a procedure commonly performed in these patients and is increasingly done at the bedside as a percutaneous dilatational technique by intensivists (3). Purported benefits of tracheostomy include assisted oral and pulmonary management, improved patient comfort, reductions in the requirement for sedation, and eased ventilator weaning (4). However, keeping tracheostomy tube for a long time may cause inflammation, impair swallowing function by impeding tracheal elevation against the epiglottis, and increases risk of adverse effects such as granulation tissue formation, tracheal stenosis, or tracheomalacia (5). It is widely accepted that there are clear benefits of decannulation (6). On the contrary, it is important to identify the influencing factors of decannulation within 6 months after tracheostomy in neurological injury patients. However, few studies had investigated the suitable predictors for successful tracheostomy decannulation. Among 131 consecutive neurosurgical patients, GCS score, the presence of vocal cord palsy, and pneumonia were reported to be associated with difficult tracheostomy decannulation within 3 months (7). Heidler and colleagues found that likelihood of successful decannulation in early rehabilitation clinics was significantly associated with age, duration of mechanical ventilation, complications, oral diet, and responsivity at admission (8). Nonetheless, predictive factor for decannulation after tracheostomy in neurological injury is still poorly understood. Meanwhile, it is important to explore an intuitive and efficient instrument which may help clinicians in estimating decannulation at early stages and optimizing their treatment strategies.

Nomogram has been accepted as a reliable tool to construct a statistical predictive model that quantifies the risk of a clinical event (9), but there is limited evidence that concentrates on the establishment of nomogram for the prediction of decannulation in patients with neurological injury. With this perspective, we conducted the current study to investigate predictive factors of decannulation within 6 months in tracheostomy-treated patients with neurological injury. With the assistance of predictive factors, we also constructed a novel nomogram model for decannulation prediction.

## Methods

### Patients and population

This study retrospectively enrolled patients with moderate or severe neurological injury, who were admitted to ICU of neurosurgical department in the First Affiliated Hospital of Nanjing Medical University between January 2016 and March 2021. Inclusion criteria are listed as follows: (1) aged ≥ 18 years; (2) time interval from symptom onset to admission ≤ 24 h; (3) patients with moderate or severe neurological injury, defined as traumatic brain injury or stroke (Glasgow Coma Scale score 9–12 and 3–8, respectively) (2); and (4) percutaneous tracheostomy performed during hospitalization. Those patients who died within 72 h of admission were excluded from the current study. Each participant or the guardian provided an informed consent form before enrollment. In addition, this study was performed with approval from the Ethics Committee of the First Affiliated Hospital of Nanjing Medical University (Reference Number 2020-SR-409).

### Data collection

After eligibility validation, one researcher collected data through a retrospective analysis of the medical records of all patients. All data were input by two research assistants and the researchers adjudicating the predictors were blind to the outcome. Data to be used in this study included (1) demographic characteristics of age, sex, smoking history, alcohol consumption history, and history of chronic diseases; (2) clinical characteristics of major diagnosis, National Institutes of Health Stroke Scale (NIHSS) score at admission, GCS score at admission, pupillary reactivity, related complications within 6 months (thoracic trauma, inhalation pneumonia, shock, intracranial infection, and epilepsy), early rehabilitation, and secondary surgery condition; and (3) tracheostomy indicators of anteropower diameter and transverse diameter of trachea, the timing of tracheostomy, time of decannulation, and related adverse events. In this study, we defined early rehabilitation as interventions directed at improving neurological impairments or disability that commence within the first 7 days after onset (10). Shock was diagnosed as the systolic blood pressure of <90 mmHg or the mean arterial pressure <70 mmHg, with associated tachycardia, tissue hypoperfusion, and hyperlactatemia (11). Secondary surgery refers to the secondary surgical treatment for recurrent cerebral hemorrhage or aggravation of hydrocephalus after the first operation (12). Tracheostomy performed in the first week from admission was defined as early tracheostomy, while later than 7 days was classified as late (13). The process of tracheostomy weaning in our institution is based on the standard decisional flowchart summarized by Ceriana et al. (14). The following criteria needed to be satisfied: (1) stable clinical condition; (2) PaCO_2_ < 60 mmHg; (3) no delirium; (4) normal endoscopic examination or revealing stenotic lesions occupying <30% of the airway; (5) patient able to expectorate on request and develop a maximal expiratory pressure of at least 40 cmH_2_O; (6) urgent swallowing evaluated by gag reflex, blue dye, and videofluoroscopy. The inner diameter of the cannula was reduced to 6 mm and kept capped for 3–4 days when all the criteria had been met. If the arterial blood gases remained constant (pH ≥ 7.35 and PaCO_2_ < 5% increase), decannulation was performed. In our study, the cannulated group was defined as long-term cannulation during the first 6 months after tracheostomy. Patients with a need for reintubation after decannulation or death during the 6-month period were also allocated as cannulated group (15). Date of decannulation was collected from inpatient/physician records. Patients were followed till discharge or till the day of decannulation.

### Statistical analysis

Continuous variables were presented as median with interquartile range or mean with standard deviation. Categorical variables were presented as counts and proportion. Differences between continuous variables were compared using *t*-test. Chi-squared test was used for categorical variables and Wilcoxon rank-sum test was applied to compare variables with a non-normal distribution. Univariate binary logistic regression analyses were performed to determine independent predictor for the probability of decannulation. Collinearity of combinations of variables was evaluated by tolerance and variation inflation factor (VIF) values, with tolerance < 0.1 and VIF > 10 considered indicative of multicollinearity (16). All variables with *P* < 0.05 in univariate logistic analyses were further assessed by multivariable logistic regression using a backward stepwise selection. Data were presented as odds ratio (OR) and 95% confidence interval (95% CI). Receiver operating characteristic curves (ROC) were constructed to evaluate predictive performance. Variables with *P* < 0.05 in multivariate analysis were incorporated into R software to construct the nomogram of the prediction model. Nomogram was subjected to 1000 bootstrap resamples for internal validation to assess predictive accuracy (17), and calibration curves were plotted to calibrate the nomogram. In a well-calibrated model, predictions should fall on a 45-degree diagonal line. In addition, Harrell's C-index was measured to quantify the discrimination performance of the nomogram, ranging from 0.5 (absence of discrimination) to 1 (perfect discrimination) (18). Finally, we plotted Kaplan–Meier curves over the tertiles of patients stratified by scores predicted by nomograms in dataset to further assess calibration and performed subgroup analysis stratified by age (<60 years old or ≥60 years old) and whether early rehabilitation was performed (19). Statistical analyses were performed in SPSS version 26.0 (IBM Corp, Armonk, NY, USA) and graphics produced with R software (Version 4.1.1.). The R packages rms, grid, lattice, Formula, and ggplot2 were used for the analysis. For all analyses, statistical significance was set at *P* < 0.05.

## Results

### Patient baseline characteristics and univariate analysis

A total of 408 patients with moderate or severe neurological injury receiving tracheostomy were admitted to ICU of neurosurgical department between January 2016 and March 2021. Forty-one patients were excluded: 6 for missing or overlapping data and 35 for lack of outcome at follow-up time point. A total sample of 367 fulfilled the inclusion criteria, of whom 69 died within 6 months. Of these tracheostomized subjects, 147 (40.1%) were decannulated within a median of 89 days (interquartile range 59–133) from tracheostomy. Patients were followed until the day of decannulation to assess for complications. Twenty-seven (19.1%) patients were diagnosed with dysarthria, 25 (17.0%) patients were detected with swallowing dysfunction, two (1.4%) with incomplete healing of incision during tracheal extubation, and 19 (12.9%) with tracheostomy-related pulmonary infection.

The univariate results of factors associated with the probability of decannulation showed that age (*P* = 0.002), GCS score (*P* < 0.001), NIHSS score (*P* < 0.001), pupillary reactivity (*P* = 0.007), early rehabilitation (*P* < 0.001), heart disease history (*P* = 0.033), history of chronic lung disease (*P* = 0.011), shock (*P* < 0.001), secondary surgery (*P* = 0.001), and transverse diameter of trachea (*P* = 0.006) were associated with decannulated at 6 months. In addition, there were no significant differences in other factors between groups (*P* > 0.05). Detailed information about the characteristics of patients is shown in Table 1.

**Table 1:** Univariate and multivariate logistic regression analyses of patients decannulated within 6 months.

**Varibales**	**Univariate**			**Multivariate**		
	**Decannulated** **(*****n*** **= 147)**	**Cannulated** **(*****n*** **= 220)**	***P*** **-value**	**OR**	**95% CI**	***P*** **-value**
Age	55.42 ± 13.98	59.89 ± 13.06	0.002	0.972	0.954–0.990	0.003
Sex (male)	93 (63.3%)	149 (67.7%)	0.377			
**Type of disease**			0.203			
Traumatic brain injury	48 (32.7%)	76 (34.5%)				
Ischemic stroke	2 (1.4%)	10 (4.5%)				
Hemorrhagic stroke	97 (66.0%)	134 (60.9%)				
GCS	6 (5,9)	5 (4,7)	<0.001			
NIHSS	34 (29,40)	39 (34,40)	<0.001	0.936	0.911–1.963	<0.001
**Pupillary reactivity**			0.007			
Both reactive	102 (69.4%)	122 (55.5%)				
One reactive	21 (14.3%)	31 (14.1%)				
Both unreactive	27 (16.3%)	67 (30.5%)				
Heart disease	17 (11.6%)	44 (20.0%)	0.033			
High blood pressure	78 (53.1%)	115 (52.3%)	0.882			
Diabetes	22 (15.0%)	41 (18.6%)	0.361			
Renal insufficiency	4 (2.7%)	12 (5.5%)	0.209			
Chronic lung disease	4 (2.7%)	21 (9.5%)	0.011	0.310	0.094–1.020	0.054
Smoker	33 (22.4%)	49 (22.3%)	0.968			
Alcohol involved	28 (19.0%)	36 (16.4%)	0.507			
Thoracic trauma	16 (10.9%)	27 (12.3%)	0.685			
Inhalation pneumonia	29 (19.7%)	37 (16.8%)	0.477			
Shock	5 (3.4%)	33 (15.0%)	<0.001	0.175	0.058–0.533	0.002
Intracranial infection	14 (9.5%)	12 (5.5%)	0.137			
Epilepsy	16 (10.9%)	24 (10.9%)	0.994			
Early rehabilitation	61 (41.5%)	31 (14.1%)	<0.001	5.062	2.889–8.868	<0.001
Secondary surgery	6 (4.1%)	34 (15.5%)	0.001	0.210	0.078–0.566	0.002
Early tracheostomy	91 (61.9%)	142 (64.5%)	0.607			
Anteropower diameter of trachea	18.16 ± 3.17	18.30 ± 3.14	0.687			
Transverse diameter of trachea	16.58 ± 2.41	17.29 ± 2.40	0.006			

### Multivariate analysis

**Figure 1:**
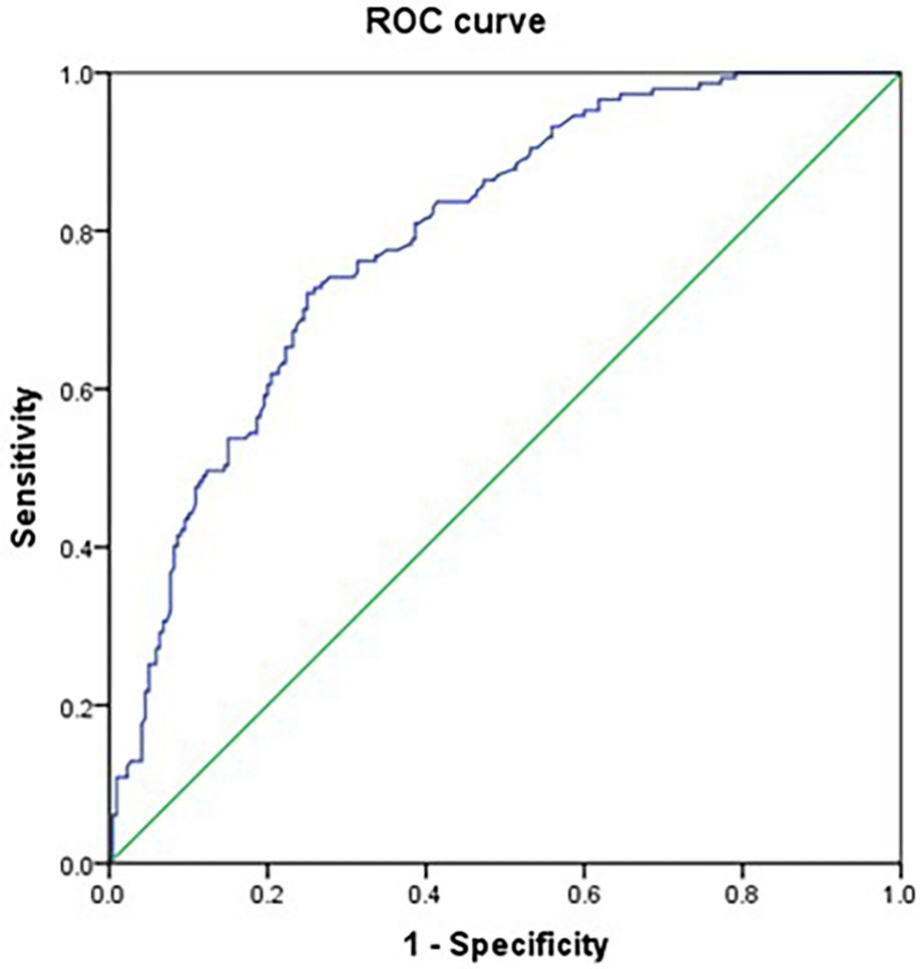
ROC curves for the probability of decannulation at 6 months.

There was significant correlation between NIHSS and GCS scores (tolerance < 1, VIF > 10); however, backward stepwise selection in the multivariate regression excluded GCS score and eliminated the bias caused by collinearity, indicating that NIHSS score was more significant for the outcome. Furthermore, the tolerance was >1, and VIF was significantly <10 for all other variables, indicating non-significant collinearity among the other independent variables. A multivariable logistic regression analysis was performed with these significant variables in univariate analysis. It revealed that age (odds ratio [OR], 0.972; 95% confidence interval [CI], 0.954–0.990; *p* = 0.003), NIHSS scores (OR, 0.936; 95% CI, 0.911–0.963; *p* < 0.001), early rehabilitation (OR, 5.062; 95% CI, 2.889–8.868; *p* < 0.001), shock (OR, 0.175; 95% CI, 0.058–0.533; *p* = 0.002), and secondary surgery (OR, 0.210; 95% CI, 0.078–0.566; *p* = 0.002) were significant predictive factors (Table 1). Receiver operating characteristic curve analysis was conducted to determine the predictability of multivariable logistic regression analysis with the results illustrated in Figure 1. The area under curve value for discriminating the probability of decannulation at 6 months was 0.793 (95% CI, 0.747–0.838; *P* < 0.001).

### Development and evaluation of the prediction model

**Figure 2:**
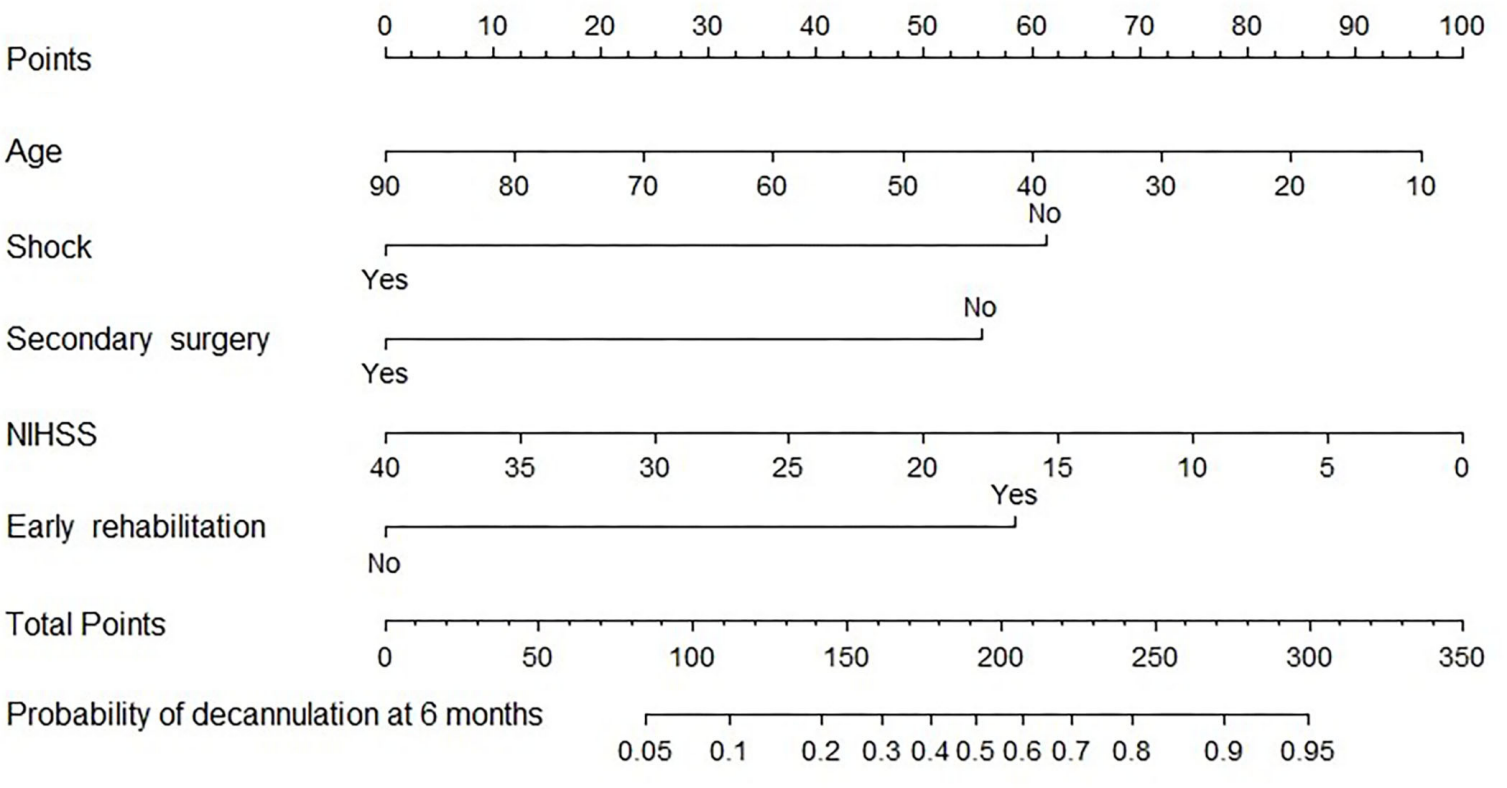
Nomogram for predicting 6-month decannulation probability. To determine the probability of decannulation at 6-month, locate the patient's age and draw a line straight up to the point's axis to establish the score associated with age. Do this again for the other four covariates (shock, secondary surgery, NIHSS, and early rehabilitation), each time drawing a straight line upward toward the point's axis. Add the scores for each covariate together and locate the total score on the total point's axis. Draw a straight line down to the lowest line to find patient's probability of decannulation at 6-month.

**Figure 3:**
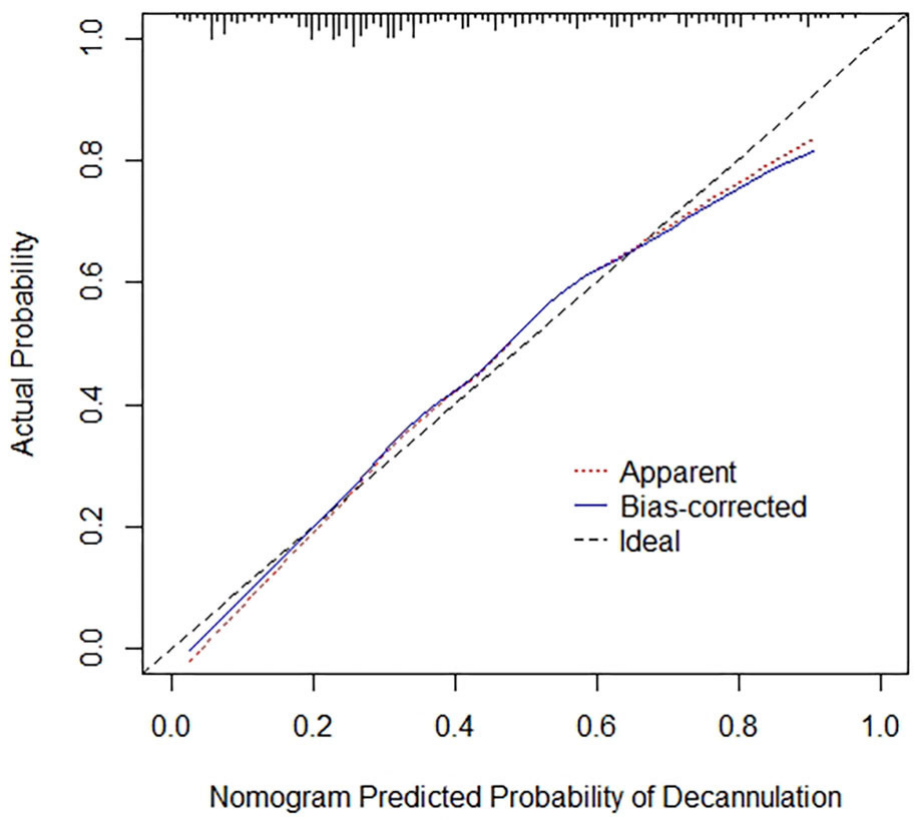
Calibration curves for nomogram. Red dotted line represents the entire cohort (*n* = 367), while blue solid line is the result after bias correction by bootstrapping (1000 repetitions), indicating nomogram performance.

A nomogram prediction model was constructed with multivariate analysis results. As shown in Figure 2, the total points represent the cumulative sum of the points of each index, and the probability represents the probability of result variable corresponding to total points. The concordance index was 0.788 by internal validation using bootstrapping with 1,000 iterations, which indicated that the predictive model had favorable discrimination. In addition, overall calibration plots were outstanding for the probability of decannulation between probabilities predicted by nomogram and actual probabilities (Figure 3).

**Figure 4:**
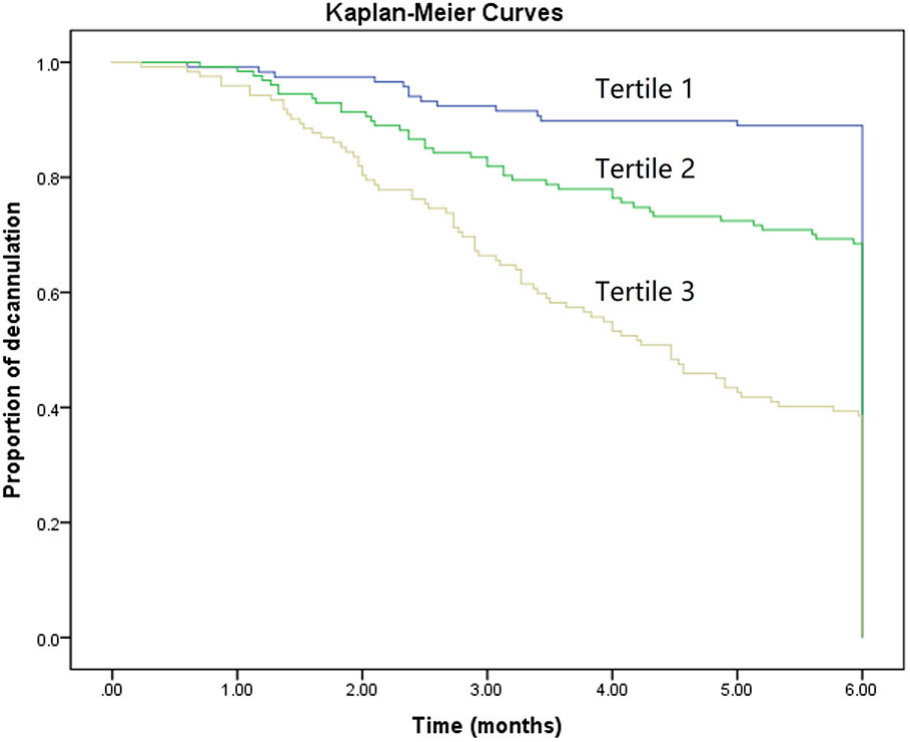
Kaplan–Meier curves demonstrating the probability of decannulation within 6 months according to tertiles of predicted scores.

To further assess the discriminative ability of model, the predicted probability of decannulation was then plotted as Kaplan–Meier curves stratified by tertile of the predicted probability (Figure 4). Patients with lowest predicted 6-month decannulation (tertile 3) had a worse outcome (14.4% 6-month decannulation) compared with patients in tertiles 1 and 2 (68.0 and 37.0% 6-month decannulation, respectively) (*P* < 0.001). Compared with actual survival based on Kaplan–Meier tables, the mean 6-month decannulation predicted time by nomogram revealed good estimation of 4.1, 5.0, and 5.6 months in tertiles 1, 2, and 3, respectively (*P* < 0.001).

**Figure 5:**
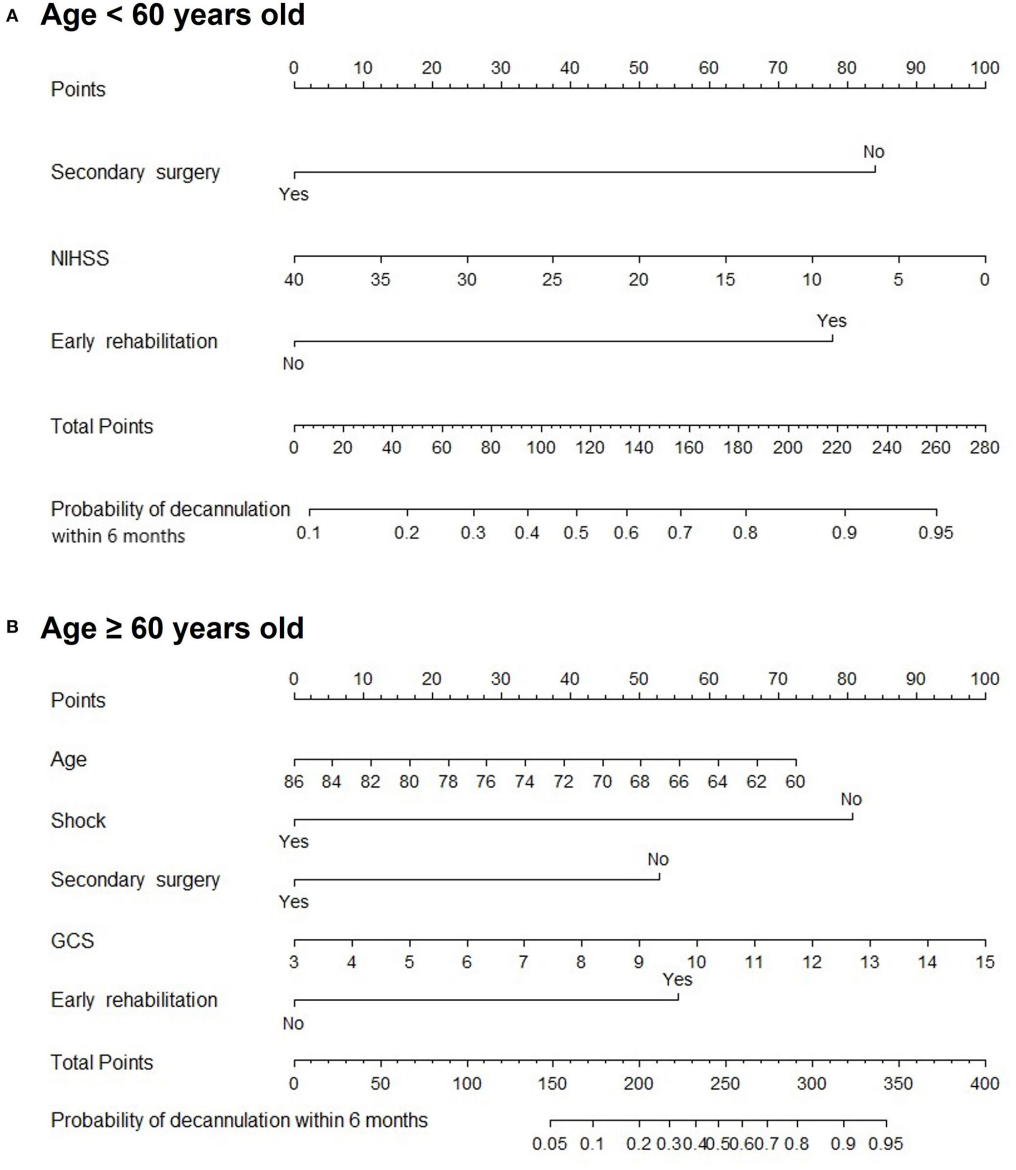
Nomogram for predicting 6-month decannulation probability in subgroup stratified by age. **(A)** Patients younger than 60 years old. **(B)** Patients aged 60 years or older.

**Figure 6:**
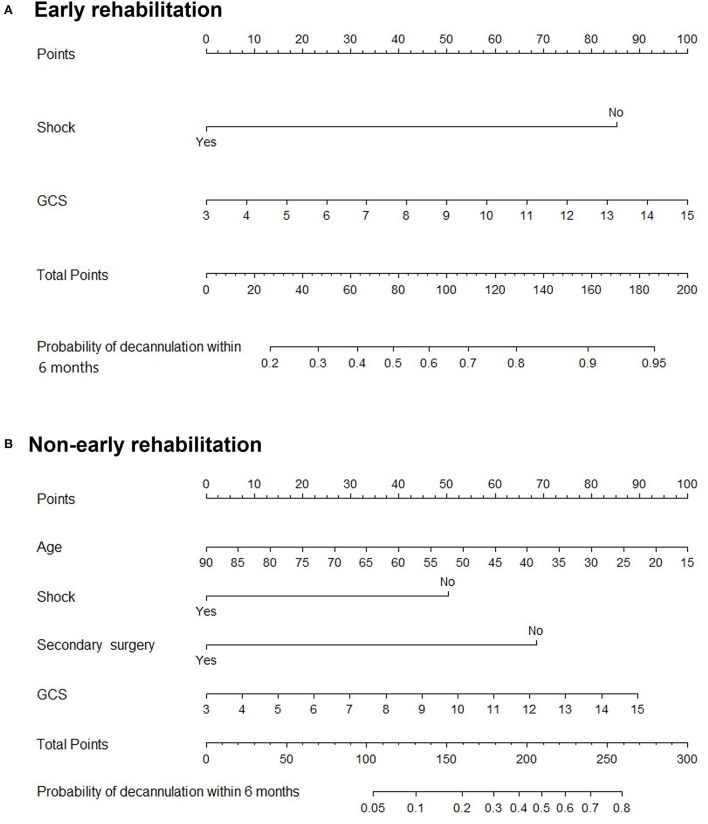
Nomogram for predicting 6-month decannulation probability in subgroup stratified by early rehabilitation. **(A)** Patients who received early rehabilitation. **(B)** Patients who not received early rehabilitation.

Subgroup analysis demonstrated that secondary surgery, NIHSS, and early rehabilitation might be associated with patients younger than 60 years (*P* < 0.05). Moreover, age, shock, secondary surgery, GCS, and early rehabilitation were significant predictive factors in patients older than 60 years, as shown in Figure 5. Figure 6 shows nomogram of subgroup analysis stratified by whether early rehabilitation was performed. Shock and GCS were identified as independent predictors for patients who received early rehabilitation. Age, shock, secondary surgery, and GCS were significantly associated with patients who not received early rehabilitation (*P* < 0.05).

## Discussion

We developed and validated a simple intuitive statistical predictive model to quantify the probability of decannulation within 6 months in patients with neurological injury treated with tracheostomy. Patients with severe neurological impairment often require long periods of tracheostomy for airway protection and mechanical ventilation (20). Clinicians are required to provide a qualified estimation on the probability of decannulation (21). Therefore, it is important to explore a clinically applicable instrument for predicting the probability of decannulation. A meta-analysis showed that half of severe acute brain injury patients were decannulated at 6–12 months (22). Hakiki et al. reported that 54.1% patients with severe brain injury could be safely decannulated during their intensive rehabilitation unit stay (23). In our study, 40.1% patients were decannulated within 6 months. The lower decannulation rate in the current study may be due to heterogeneity of primary diseases including traumatic brain injury, ischemic stroke, and hemorrhagic stroke, while other studies mainly focused on patients with traumatic brain injury, especially for those presenting consciousness.

Our model used ascertainable clinical and pathological risk factors to conduct prediction on decannulation, including age, NIHSS scores, early rehabilitation, shock, and secondary surgery. Age as an inverse predictor of decannulation was included in the model. This variable was in line with almost all previous prognostic studies investigating predictors of decannulation in either subjects with acquired brain injury or critically ill subjects (8, 24, 25). In general, younger patients had a higher probability of decannulation than older patients.

NIHSS is the most widely used scale for evaluating the severity of stroke in the world. At present, the initial NIHSS score is commonly used as an appealing prognostic marker for long-term functional outcome of stroke patients (26). In this study, NIHSS scores may serve as a better prognostic marker of severe neurological injury in hospitalization. Mortensen et al. (27) developed a prognostic model for decannulation in patients with acquired brain injury, which included overall functional level measured with Early Functional Abilities (EFA) score. In our study, GCS was associated with the probability of decannulation in univariate analyses but not in multivariate analyses. Clinicians usually use GCS to evaluate nervous system functional defects of acute stroke patients, such as difficulty in eye closure, decerebration, and decortication symptoms. For patients with stroke, GCS often overestimates degree of neurological deficit. Therefore, it may be unsuitable for stroke patients without impaired consciousness (28).

Early rehabilitation as a predictor variable was an important clinical indicator, which was previously highlighted by Zivi et al. (29). They demonstrated that an early neurorehabilitation protocol carried out during ICU period could increase chance of decannulation by more than 300% for tracheostomized patients affected by acquired brain injury. Vitacca et al. (30) also confirmed the relationship between physiotherapy and probability of decannulation. They found that eighty-three% of critical care survivors underwent physical exercise were decannulated vs. 14% of controls. Acute neurorehabilitation unit is another option for assisting tracheostomy decannulation, in which their decannulation failure rate significantly reduced from 27.3 to 9.1% (31). Therefore, data obtained in our single center reinforced the evidence that early rehabilitation, which included progressive peripheral muscle physiotherapy, respiratory exercise, neuromuscular electronic stimulation (NMES), speech, and swallowing training, was feasible for tracheostomized neurological injury patients. Indeed, several reasons, such as clinical instability and inability to cooperate, often preclude some patients from participating (32).

Subgroup analysis stratified by age showed that there was no significant association between age and decannulation outcome in patients younger than 60 years old. However, in the subgroup of patients older than 60 years old, the probability of decannulation decreased with increasing age, which may be related to the decline of cardiopulmonary reserve capacity in elderly populations (33). On the contrary, the difference in the occurrence of shock in the age subgroup may be due to the small effect of the correction of circulatory shock on organ function in the young population. However, shock in elderly population has a great impact on organ function and is more likely to leave related multiple organ dysfunction (34). The overall nomogram indicates the positive significance of early rehabilitation for successful decannulation in patients with moderate/severe neurological injury within 6 months. Early rehabilitation is worth popularizing in clinical practice, but some grass-roots hospitals lack early rehabilitation intervention conditions. Therefore, we set up subgroups and drew nomograms according to whether early rehabilitation was performed which can be applied to different medical institutions to predict 6-month decannulation of patients with severe neurological injury. While the small size of some subgroups limits generalizability, our results highlight a need for larger studies providing more outcome data for each condition separately.

To the extent of our knowledge, no study tackled the prediction of decannulation probability using data available by nomogram models. Our model was developed in a moderate or severe neurological injury cohort and focused on demographic and clinical variables that would be routinely available at time of treatment. The present nomogram was comprised of five variables: age, NIHSS scores, whether early rehabilitation was performed, whether shock occurred, and whether secondary surgery was performed during hospitalization. These factors have been incorporated into a nomogram to assist clinicians in estimating the probability of decannulation for individual patients, which in turn may assist with medical decision (35). Our nomogram model showed that using the above independent risk factors as predictive variables had a good C-index level and a good correlation with actual occurrence. Correction curves with internal validation using bootstrapping with 1,000 iterations also showed that our nomogram model could effectively predict the probability of decannulation within 6 months in patients with moderate or severe brain injury receiving tracheostomy.

However, this study also has the following shortcomings. First, our study excluded patients who died within 72 h and included only patients with traumatic brain injury and stroke, which limited the wider application of the model. Second, we constructed prediction nomogram based on the retrospective review of medical records, and the database did not include other risk factors for neurological injury such as swallowing function and laboratory markers. Thus, restricted in using certain factors may have limited the power of our nomogram to identify their significance. Finally, since the present study was conducted at a single center, our findings may have limited generalizability and additional external validations; therefore, larger samples are warranted to direct implementation of our model in clinical practice.

## Conclusion

Multiple clinical parameters have been shown to have prognostic accuracy for decannulation in patients with moderate/severe neurological injury, and further investigation is warranted. We developed a nomogram model for the prediction of decannulation with the predictors including age, NIHSS scores, presence of early rehabilitation, presence of shock, and presence of secondary surgery, which may help clinicians in estimating patients' prognosis.

## Data availability statement

The raw data supporting the conclusions of this article will be made available by the authors, without undue reservation.

## Ethics statement

The studies involving human participants were reviewed and approved by the Ethics Committee of the First Affiliated Hospital of Nanjing Medical University. The patients/participants provided their written informed consent to participate in this study.

## Author contributions

XW, LW, and ZW conceived and designed the study. XW, YS, XL, and FL collected the dates. LW analyzed the results and wrote the manuscript. YZ reviewed and edited the manuscript. All authors contributed to the article and approved the submitted version.

## Conflict of interest

The authors declare that the research was conducted in the absence of any commercial or financial relationships that could be construed as a potential conflict of interest.

## Publisher's note

All claims expressed in this article are solely those of the authors and do not necessarily represent those of their affiliated organizations, or those of the publisher, the editors and the reviewers. Any product that may be evaluated in this article, or claim that may be made by its manufacturer, is not guaranteed or endorsed by the publisher.
